# Plasmid-mediated colistin resistance among human clinical *Enterobacterales* isolates: national surveillance in the Czech Republic

**DOI:** 10.3389/fmicb.2023.1147846

**Published:** 2023-04-27

**Authors:** Marketa Zelendova, Costas C. Papagiannitsis, Petra Sismova, Matej Medvecky, Katarina Pomorska, Jana Palkovicova, Kristina Nesporova, Vladislav Jakubu, Ivana Jamborova, Helena Zemlickova, Monika Dolejska, Working Group for Monitoring of Antibiotic Resistance

**Affiliations:** ^1^Department of Biology and Wildlife Diseases, Faculty of Veterinary Hygiene and Ecology, University of Veterinary Sciences Brno, Brno, Czechia; ^2^CEITEC VETUNI, University of Veterinary Sciences Brno, Brno, Czechia; ^3^Department of Microbiology, University Hospital of Larissa, Larissa, Greece; ^4^Department of Chemistry, Faculty of Science, University of Hradec Králové, Hradec Králové, Czechia; ^5^NRL for ATB, The National Institute of Public Health, Centre for Epidemiology and Microbiology, Prague, Czechia; ^6^Department of Microbiology, 3rd Faculty of Medicine, Kralovske Vinohrady University Hospital and National Institute of Public Health, Charles University, Prague, Czechia; ^7^Department of Clinical Microbiology and Immunology, Institute of Laboratory Medicine, The University Hospital Brno, Brno, Czechia; ^8^Department of Microbiology, Faculty of Medicine and University Hospital in Plzen, Charles University, Pilsen, Czechia

**Keywords:** antibiotic resistance, *Enterobacterales*, human, *mcr*, plasmids

## Abstract

The occurrence of colistin resistance has increased rapidly among *Enterobacterales* around the world. We performed a national survey of plasmid-mediated colistin resistance in human clinical isolates through a retrospective analysis of samples from 2009 to 2017 and a prospective sampling in 2018–2020. The aim of this study was to identify and characterize isolates with *mcr* genes from various regions of the Czech Republic using whole genome sequencing (WGS). Of all 1932 colistin-resistant isolates analyzed, 73 (3.8%) were positive for *mcr* genes. Most isolates carried *mcr-1* (48/73) and were identified as *Escherichia coli* (*n* = 44) and *Klebsiella pneumoniae* (*n* = 4) of various sequence types (ST). Twenty-five isolates, including *Enterobacter* spp. (*n* = 24) and *Citrobacter freundii* (*n* = 1) carrying the *mcr-9* gene were detected; three of them (*Enterobacter kobei* ST54) co-harbored the *mcr-4* and *mcr-9* genes. Multi-drug resistance phenotype was a common feature of *mcr* isolates and 14% (10/73) isolates also co-harbored clinically important beta-lactamases, including two isolates with carbapenemases KPC-2 and OXA-48. Phylogenetic analysis of *E. coli* ST744, the dominant genotype in this study, with the global collection showed Czech isolates belonged to two major clades, one containing isolates from Europe, while the second composed of isolates from diverse geographical areas. The *mcr-1* gene was carried by IncX4 (34/73, 47%), IncHI2/ST4 (6/73, 8%) and IncI2 (8/73, 11%) plasmid groups. Small plasmids belonging to the ColE10 group were associated with *mcr-4* in three isolates, while *mcr-9* was carried by IncHI2/ST1 plasmids (4/73, 5%) or the chromosome (18/73, 25%). We showed an overall low level of occurrence of *mcr* genes in colistin-resistant bacteria from human clinical samples in the Czech Republic.

## Introduction

The excessive consumption of antimicrobial substances associated with faster spread of antibiotic resistance represents a global concern. The dissemination of multi-drug resistant (MDR) bacteria resulted in limited treatment options of infectious diseases in healthcare systems. The interest in the administration of older antibiotics such as polymyxins has therefore been renewed (Bitar et al., 2020). Colistin has been widely used in the past, but due to its nephrotoxicity and neurotoxicity, it has become a restricted antibiotic (Viñes et al., 2021). Currently, colistin is administered for the treatment of life-threatening infections caused by MDR Gram-negative pathogens as the last-resort antibiotic (Yilmaz et al., 2016; Hamel et al., 2021; Viñes et al., 2021). In contrast, colistin has been widely used for prophylactic and therapeutic purposes in veterinary medicine for decades (Quiroga et al., 2019; Viñes et al., 2021). However, colistin overuse in livestock has led to the spread of colistin-resistant pathogens worldwide and the development of different strategies used by bacteria to increase resistance against colistin (El-Sayed Ahmed et al., 2020).

Resistance to colistin can be either associated with chromosomal mutations or with *mcr* genes carried by plasmids that are facilitating horizontal transfer of colistin resistance between bacteria (Zhu et al., 2019). Acquired colistin-resistance mechanisms have been recognized in some members of the Enterobacteriaceae family, such as *E. coli, Salmonella* spp., *Klebsiella* spp., and *Enterobacter* spp. These include genes and operons responsible for encoding enzymes that have a direct role in LPS modification, such as the *pmrC* and *pmrE* genes and the *pmrHFIJKLM* operon (Aghapour et al., 2019). Apart from the chromosomally-mediated mechanisms, 10 variants, *mcr-1* to *mcr-10*, carried by various plasmid families have been so far identified in *Enterobacterales*, especially in *E. coli* and *Enterobacter* spp. (Bitar et al., 2020; Javed et al., 2020; Li et al., 2020; Wang C. et al., 2020). The most common variant, *mcr-1*, is usually located on plasmids of various incompatibility (Inc) groups, but predominantly on IncX4, IncI2 and IncHI2 (Carattoli et al., 2014; Doumith et al., 2016; Zelendova et al., 2021). These plasmid types carrying *mcr-1* were found in *Enterobacterales* isolates from humans as well as farm animals around the globe (Dalmolin et al., 2018; Quiroga et al., 2019), highlighting their wide distribution in various niches. Besides *mcr-1*, other genes for plasmid-mediated colistin resistance, such as *mcr-4* and *mcr-9*, have been reported (Bitar et al., 2020; Li et al., 2020). The *mcr-4* gene is usually located on small ColE10-type plasmids (Carattoli et al., 2017; Marchetti et al., 2021) while *mcr-9* is mostly carried by large IncHI2 plasmids or is incorporated into the chromosome (Li et al., 2020; Tyson et al., 2020).

The emergence of colistin resistance in MDR bacteria is a significant clinical concern. Isolates encoding extended-spectrum beta-lactamase (ESBL) or carbapenemase on a single plasmid along with *mcr* have been detected (Carattoli et al., 2014; Katip et al., 2021). As the co-occurrence of more resistance genes within the bacteria represents a threat to current medicine, The European Centre for Disease Prevention and Control (ECDC) published the expert protocol that recommends performing the surveillance of co-resistance to both colistin and carbapenems in *Enterobacterales* (ECDC, European Centre for Disease Prevention and Control, 2019).

From the Czech Republic, only sporadic reports describing the identification of *mcr*-carrying isolates in clinical samples have been published so far (Bitar et al., 2019; Bitar et al., 2020; Krutova et al., 2021). However, overview data on the prevalence of *mcr* genes in Czech patients are not available. To fill in this gap, we aim to identify *mcr* genes in colistin-resistant human clinical isolates of Gram-negative bacteria from the Czech Republic between 2009 and 2020, and to determine characteristics of the *mcr*-positive strains using whole genome sequencing (WGS), plasmid typing and transferability experiments.

## Materials and methods

### Sampling and detection of *mcr* genes

A total of 1932 colistin-resistant isolates of Gram-negative bacteria with a minimum inhibitory concentration (MIC) to colistin >2 mg/L collected from Czech patients between 2009 and 2020 were examined. The collection consisted of 682 retrospective isolates obtained from 2009 through 2017 during various surveillance programmes at the National Institute of Public Health that were not targeting colistin resistance. The retrospective collection included mainly *Klebsiella pneumoniae* (*n* = 429), *Enterobacter* spp. (*n* = 108), *Pseudomonas aeruginosa* (*n* = 49), *Acinetobacter baumanii* (*n* = 33), *Stenotrophomonas maltophilia* (*n* = 20), *Escherichia coli* (*n* = 14) and 29 isolates of other 13 species. Prospective surveillance of colistin resistance in Czech hospitals was carried out during 2.5-year period between January 2018 and June 2020. It resulted in the collection of 1,250 isolates of *Klebsiella pneumoniae* (*n* = 491), *Enterobacter* spp. (*n* = 311), *Escherichia coli* (*n* = 179), *Pseudomonas aeruginosa* (*n* = 99), *Acinetobacter baumanii* (*n* = 43), *Hafnia alvei* (*n* = 28), *Klebsiella variicola* (*n* = 20), *Acinetobacter* spp. (*n* = 15), *Salmonella enterica* (*n* = 15), *Klebsiella oxytoca* (*n* = 14), *Klebsiella aerogenes* (*n* = 10), and 25 isolates of other 11 species. Strain identification was performed by a matrix-assisted laser desorption ionization-time of flight mass spectrometer (MALDI-TOF) using MALDI Biotyper software (Bruker Daltonics, Bremen, Germany). Species-level discrimination of *Enterobacter* spp. was performed using average nucleotide identity (ANI) (Yoon et al., 2017) and digital DNA–DNA hybridization (Meier-Kolthoff et al., 2013) of whole genome sequences. Eight types of strains were used as reference species, including *Enterobacter asburiae* (ATCC35953^T^), *Enterobacter bugandensis* (DSM 29888^T^), *Enterobacter cloacae* (ATCC13047^T^), *Enterobacter dykesii* (DSM111347^T^), *Enterobacter hormaechei* (ATCC49162^T^), *Enterobacter kobei* (DSM13645^T^), *Enterobacter vonholyi* (DSM110788^TT^), *Enterobacter roggenkampii* (DSM16690^T^).

All isolates were subjected to multiplex polymerase chain reaction (PCR) to detect the variants of *mcr* genes (*mcr-1* to *mcr-9*) (Rebelo et al., 2018; Wang et al., 2018; Kieffer et al., 2019).

### Antimicrobial susceptibility testing

Susceptibility profiles of *mcr*-positive isolates were determined by the broth microdilution method using the following 15 antimicrobial substances: amikacin, ampicillin, ampicillin/sulbactam, cefepime, cefotaxime, cefoxitin, ceftazidime, ceftolozane/tazobactam, colistin, cotrimoxazole, ciprofloxacin, gentamicin, meropenem, piperacillin/tazobactam and tobramycin. The production of ESBL and AmpC type beta-lactamase was tested by a double-disk synergy test (European Committee on Antimicrobial Susceptibility Testing, 2017). The production of carbapenemase was tested by a combination disc test method (European Committee on Antimicrobial Susceptibility Testing, 2017) and biochemical tests (BioRad-Beta-Carba test), while carbapenem hydrolysis was tested by MALDI-TOF (Papagiannitsis et al., 2015).

### Conjugative transfer of *mcr* genes

Conjugation assays were performed to determine the transferability of *mcr* genes into plasmid-free sodium azide-resistant *E. coli* J53 K12 recipient cells using the filter-mating method (Borowiak et al., 2019). The transconjugants (TCs) were selected on LB agar plates (LBA) with sodium azide (100 mg/L) and colistin (0.5 mg/L). Successful transfer of the plasmid-mediated colistin resistance *via* conjugation was confirmed by PCR targeting the *mcr* gene (Rebelo et al., 2018; Wang et al., 2018; Kieffer et al., 2019) and *E. coli* J53 K12 (Bauer et al., 2007). The size and number of plasmids transferred were estimated by pulsed-field gel electrophoresis (PFGE) using S1 nuclease (Centers for Disease Control and Prevention, 2004) and PCR-based replicon typing (PBRT; Carattoli et al., 2005).

### Whole genome sequencing and plasmid characterization

Genomic DNA of all *mcr*-positive isolates was extracted using the NucleoSpin^®^ Tissue Kit (Macherey-Nagel GmbH & Co, Duren, Germany). The libraries were prepared using the Nextera XT DNA Sample Preparation Kit and sequenced on the MiSeq or NovaSeq 6,000 platform (Illumina, San Diego, CA, United States). Raw reads were quality- and adaptor-trimmed using Trimmomatic v0.39 (Bolger et al., 2014) and assembly was performed by SPAdes v3.12.0 (Bankevich et al., 2012) and assembled data were analyzed using the CGE tools^1^ that were used to identify antibiotic resistance genes (ResFinder 4.1) (Zankari et al., 2012), multi-locus sequence types (MLST 2.0) (Larsen et al., 2012), plasmid replicons (PlasmidFinder 2.1) and plasmid sequence types (STs) (pMLST 2.0) (Carattoli et al., 2014). Chromosomal mutations for resistance to fluoroquinolones and colistin in *E. coli* and *K. pneumoniae* isolates were determined by PointFinder (Zankari et al., 2017). Sequences of six IncX4 plasmids carrying *mcr-1* were extracted from Illumina data and gaps were filled by a PCR-based strategy and Sanger sequencing.

A complete nucleotide sequence of 12 selected isolates was obtained using long-read sequencing on the MinION platform (Oxford Nanopore Technologies, ONT, Oxford, United Kingdom). Genomic DNA was extracted by Genfind V3 (Beckman Coulter, United States). Libraries were constructed using a SQK-RBK004 rapid barcoding 1D kit according to the manufacturer’s protocol. The barcoded library mix was loaded onto a flow cell (FLO-MIN106 R9.4 SpotON) and sequenced for 48 h. The raw electrical signals were basecalled using Guppy v4.2.2 (ONT) and raw reads in fastq format were obtained. BBDuk^2^ and Porechop v0.2.4 (ONT) were used for adaptor and quality trimming (*Q* ≤ 7) and for demultiplexing, respectively. Whole plasmid sequences were assembled using Unicycler v0.4.8 (Wick et al., 2017) and Flye v2.6 (Lin et al., 2016) and polished by Illumina reads using Pilon v1.23 (Walker et al., 2014). For sequence analysis and annotation, BLAST,^3^ the ISfinder database, and the open reading frame (ORF) finder tool^4^ were used. Comparative genome alignment with corresponding reference plasmids was performed using Mauve v.2.3.1 (Darling et al., 2010). Figures were generated from sequence data using BRIG v.0.95 (Alikhan et al., 2011) and clinker v0.0.23 (Gilchrist and Chooi, 2021).

### Phylogenetic analysis

In total, four different datasets were subjected to phylogenetic analysis. Two of them were local phylogenetic trees including only isolates from our collection: the first one comprised all detected *mcr*-carrying *E. coli* isolates and the second one showed the phylogeny of *Enterobacter* spp. genomes. The third tree was global and comprised genomes of 449 *E. coli* ST744 isolates that were available at EnteroBase in April 2021^5^ along with 10 ST744 isolates from our collection. These three trees were generated based on a core-genome determined employing a Roary pipeline v3.12.0 (Page et al., 2015) and aligned with MAFFT v7.313 (Katoh and Standley, 2013). Trees were inferred under the GTR + CAT model using FastTree v2.1.11 (Price et al., 2010) compiled with double precision arithmetic.

Remaining detailed tree topology was constructed based on a pipeline described in a previous study (Forde et al., 2022) using Python scripts that are available on GitHub.^6^ Based on the *E. coli* ST744 global tree, 38 Illumina SRA archives belonging to isolates that were closely related to 10 ST744 isolates from our collection were gathered from the GenBank database in May 2021. Raw sequencing reads of those 38 isolates along with another 10 from our collection were subjected to quality trimming *via* Trimmomatic tool v0.36 (Bolger et al., 2014) and consequently mapped to *E. coli* str. K-12 substr. MG1655 reference genome (GenBank accession U00096.3) using Bowtie2 v2.3.4.2 (Langmead and Salzberg, 2012). Single nucleotide polymorphisms (SNPs) were detected in individual isolates by VarScan v2.4.4 (Koboldt et al., 2012) using the following parameters: minimum read depth of 8; minimum base quality of 20; variant allele frequency ≥ 0.80. Problematic sites were then removed based on the following rules: occurred in phage regions as detected by PHASTER (Arndt et al., 2016); occurred in repetitive/homologous genomic regions; more than 5 isolates at a particular site showed prevalent base frequency below 80% or/and read depth below 8. The resulting alignment file was then subjected to maximum-likelihood analysis using RAxML v8.2.11 (Stamatakis, 2014) under the GTR + CAT model of nucleotide substitution with 500 rapid bootstrap replicates using sample SRR9990292 as an outgroup. Tree topologies were visualized *via* iTOL v6.3 (Letunic and Bork, 2021) and edited using Inkscape v1.1.^7^ The SNP distance matrix was generated by snp-dists 0.7.0 software.^8^

### Nucleotide sequence accession numbers

Genome assemblies, SRA archives and annotated plasmid sequences (Table 1) were deposited in the NCBI under BioProject with accession number PRJNA772899.

**Table 1:** The characteristics of sequenced *mcr*-encoding plasmids.

Plasmid name	Organism (ST)^1^	Plasmid-carrying *mcr*^2^	*mcr*	Other ARGs in *mcr* plasmid^3^	WGS platform	Accession no.
pMCR1-40331	*K. pneumoniae* (ST290)	IncX4 (33,303)	*mcr-1.1*	–	Illumina	OP428973
pMCR1-42913	*E. coli* (ST448)	IncX4 (33,304)	*mcr-1.1*	–	Illumina	OP428974
pMCR1-44158	*E. coli* (ST156)	IncX4 (33,303)	*mcr-1.2*	–	Illumina	OP428975
pMCR1-44653	*E. coli* (ST1196)	IncX4 (33,303)	*mcr-1.1*	–	Illumina	OP428976
pMCR1-45082	*E. coli* (ST744)	IncX4 (34,068)	*mcr-1.1*	–	Illumina	OP428977
pMCR1-46049	*K. pneumoniae* (ST147)	IncX4 (33,303)	*mcr-1.1*	–	Illumina	OP428978
pMCR1-53288	*E. coli* (ST538)	IncI2 (60,733)	*mcr-1.1*	–	MinION	OP434482
pMCR1-43934	*E. coli* (ST8186)	IncHI2/ST4 (225,732)	*mcr-1.1*	*aph(6)-Id, aph(3)-Ib, catA1, tet*(A)	MinION	OP950834
pMCR1-51133	*E. coli* (ST117)	IncHI2/ST4 (237,743)	*mcr-1.1*	*aadA1, aadA2b, bla*_TEM-1B_*, catA1, cmlA1, qacE, sul1, tet*(A)	MinION	OP950835
pMCR1-59496	*K. pneumoniae* (ST726)	IncHI2/ST3 (254,909)	*mcr-1.1*	*aac(3)-IV, aadA1, aadA2b aph(3′)-Ia, aph(4)-Ia, bla*_CTX-M-14_*, cmlA1, floR, fosA3, mph*(A)*, sul2, sul3*	MinION	OP950836
pMCR9-16539	*E. kobei* (ST591)	IncHI2/ST1 (285,283)	*mcr-9.1*	*aadA2b, aph(3″)-Ib, aph(6)-Id, bla*_TEM-1B_*, bla*_SHV-12_*, catA2, dfrA19, qacE, qnrA1, sul1, tet*(D)	MinION	OP950838
pMCR9-17620	*E. hormaechei* (ST91)	IncHI2/ST17 (276,870)	*mcr-9.1*	*aadA2b, ant(2″)-Ia, bla*_CTX-M-9_ *, catA1, dfrA16, qacE, qnrA1, sul1*	MinION	OP950833
pMCR9-57185	*C. freundii* (ST18)	IncHI2/ST1 (330,692)	*mcr-9.1*	*aac(6′)-IIc, bla_TEM-1B_, bla*_DHA-1_*, catA2, ere*(A)*, qacE, qnrB4, sul1, tet*(D)	MinION	OP950837
pMCR4-26153	*E. kobei* (ST54)	ColE10 (12,808)	*mcr-4.2*	–	MinION	OP428979

1 ST, sequence type.

2 Plasmid carrying mcr include the information on plasmid incompatibility group (Inc), ST (if available) and plasmid size in bp.

3 ARGs, antibiotic resistance genes.

### Statistical analysis

The statistical significance of the increasing prevalence of *mcr* genes between 2018 and 2020 was calculated using Fisher’s exact test (two tailed) using a significance level *α* = 0.05.

## Results

### *mcr-*positive *Enterobacterales* isolates

From all 1932 examined colistin-resistant isolates, 73 (3.8%) were identified to carry *mcr* genes (Supplementary Table S1). Most (65/73) isolates were detected during the prospective years, including eight isolates in 2018 (3%, *n* = 274), 27 isolates in 2019 (4%, *n* = 634) and 30 isolates in 2020 (9%, *n* = 342). The difference in increasing prevalence of *mcr* genes between 2018 and 2020 was statistically significant (*p* < 0.007). From the retrospective analysis using a collection of isolates at the National Reference Laboratory for Antibiotics, seven isolates (1%, *n* = 682) carrying *mcr* genes were found, including seven *Enterobacter* spp. from 2010 (*n* = 1), 2012 (*n* = 3), 2013 (*n* = 1), 2014 (*n* = 2) and one isolate of *K. pneumoniae* (*n* = 1) from 2017. Isolates carrying *mcr-1* were identified as *E. coli* (*n* = 44) and *K. pneumoniae* (*n* = 4), while the remaining 25 isolates carried the *mcr-9.1* allele. Three of the *mcr-9.1*-carrying isolates were also positive for *mcr-4.2*/*mcr-4.3*. The isolates carrying *mcr-9.1* were identified as *Citrobacter freundii* (*n* = 1), *Enterobacter asburiae* (*n* = 13), *Enterobacter kobei* (*n* = 6), *Enterobacter cloacae* (*n* = 3). *Enterobacter roggenkampii* (*n* = 1) and *Enterobacter hormaechei* (*n* = 1). Plasmid-mediated colistin resistance genes were the most common among *E. coli* as 19% (44/231) colistin-resistant isolates carried *mcr-1*, while the occurrence in other species was rare (4.4% in *Enterobacter* spp., 0.4% in *K. pneumoniae*).

Colistin-resistant isolates carrying *mcr-1* (48/73, 66%) showed phenotypic resistance to beta-lactam antibiotics including ampicillin (46/48, 96%), ampicillin/sulbactam (43/48, 90%), cefoxitin (9/48, 19%), piperacillin/tazobactam (9/48, 19%), cefotaxime (5/48, 10%), ceftazidime (5/48, 10%), cefepime (4/48, 8%) and ceftolozane/tazobactam (2/48, 4%). Resistance to other antimicrobials including cotrimoxazole (34/48, 71%), ciprofloxacin (34/48, 71%), trimethoprim (34/48, 71%), gentamicin (11/48, 23%), tobramycin (10/48, 21%) and amikacin (1/48, 2%) was found.

The majority of colistin-resistant isolates carrying *mcr-9* were resistant to cefoxitin (25/25, 100%), ampicillin (23/25, 92%), ampicillin/sulbactam (23/25, 92%) and cefotaxime (12/25, 48%). Furthermore, they showed resistance to ceftazidime (9/25, 36%), cotrimoxazole (6/25, 24%), ciprofloxacin (5/25, 20%), tobramycin (5/25, 20%), trimethoprim (5/25, 20%), piperacillin/tazobactam (7/25, 28%), gentamicin (4/25, 16%), cefepime (3/25, 12%), amikacin (2/25, 8%), ceftolozane/tazobactam (2/25, 8%) and meropenem (2/25, 8%).

Nine isolates, including *E. coli* (*n* = 4), *Enterobacter* spp. (*n* = 3), *Citrobacter freundii* (*n* = 1) and *K. pneumoniae* (*n* = 1) with resistance to seven or more different antibiotics were simultaneously positive for ESBL production. AmpC beta-lactamase was detected in six *Enterobacter* spp. isolates and one *E. coli* isolate.

### Analysis of WGS results

**Figure 1:**
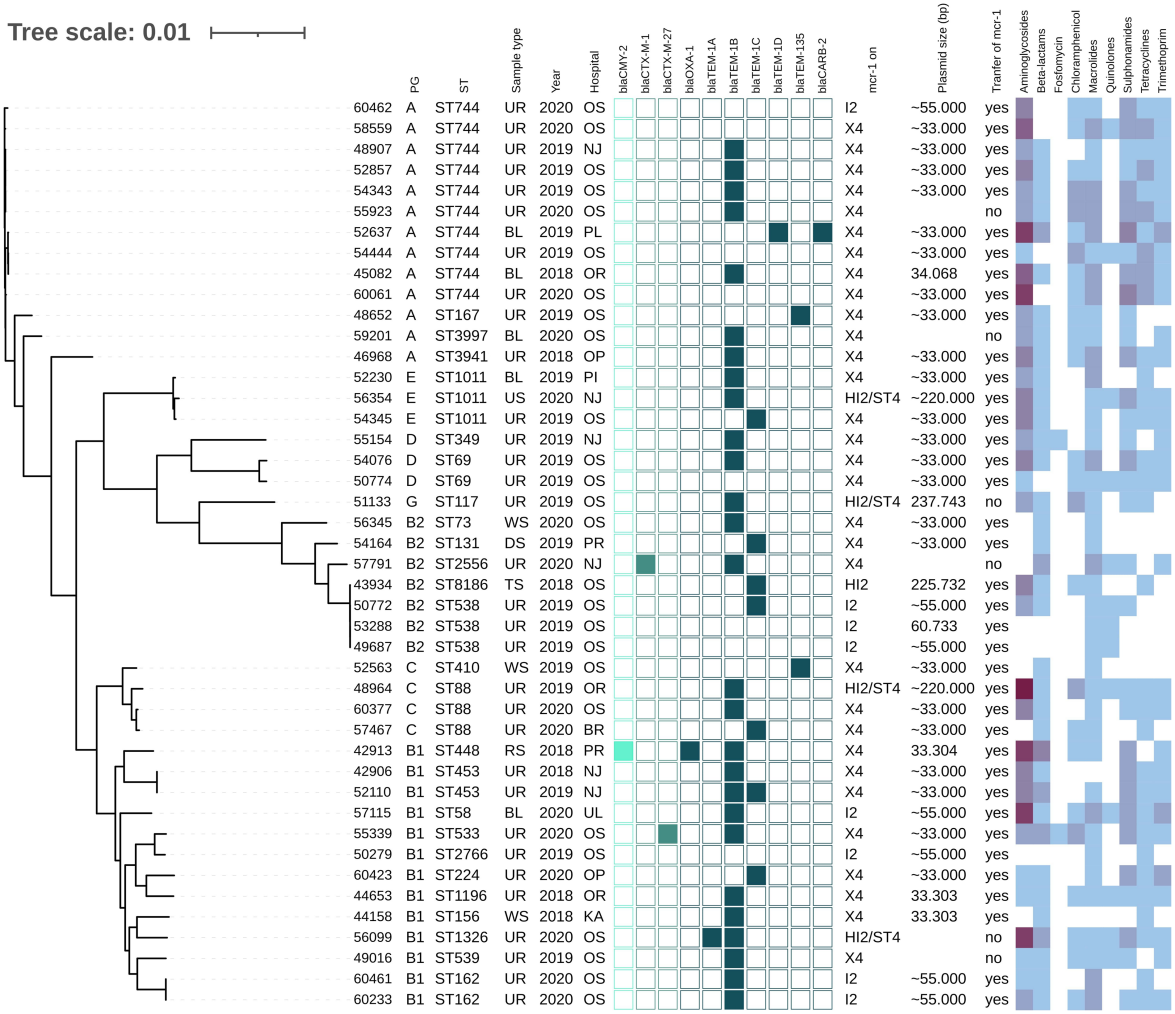
Phylogenetic tree of *E. coli* isolates with *mcr-1* of Czech clinical origin. The metadata in columns represents phylogenetic group (PG); sequence type (ST); type of sample (Sample type): urine (UR), blood (BL), rectal swab (RS), tonsil swab (TS), wound swab (WS), decubitus swab (DS), urethra swab (US); year of isolation (Year) and city where is the hospital related to the isolate recovery (Hospital): Novy Jicin (NJ), Prague (PR), Ostrava (OS), Karvina (KA), Ostrava-Poruba (OR), Opava (OP), Pribram (PI), Plzen (PL), Usti nad Labem (UL), Brno (BR). The turquoise squares represent presence (full square) or absence (empty square) or respective beta-lactamase encoding genes divided as AmpC (bright turquoise), ESBL (medium) and narrow-spectrum beta-lactamases (dark). The next section (mcr-1 on) reveals which plasmid carried *mcr-1* gene; the size of the plasmid (Plasmid size) in bp while approx. Sizes (~) are estimated based by S1-PFGE, while the more precise values come from plasmid sequencing; the success of conjugative transfer is indicated (Transfer of mcr-1). The heat map in the last section indicated the amount of antibiotic resistance genes carried by the respective isolate in specified category of antibiotics from zero (white) to maximum of six (dark purple).

**Figure 2:**
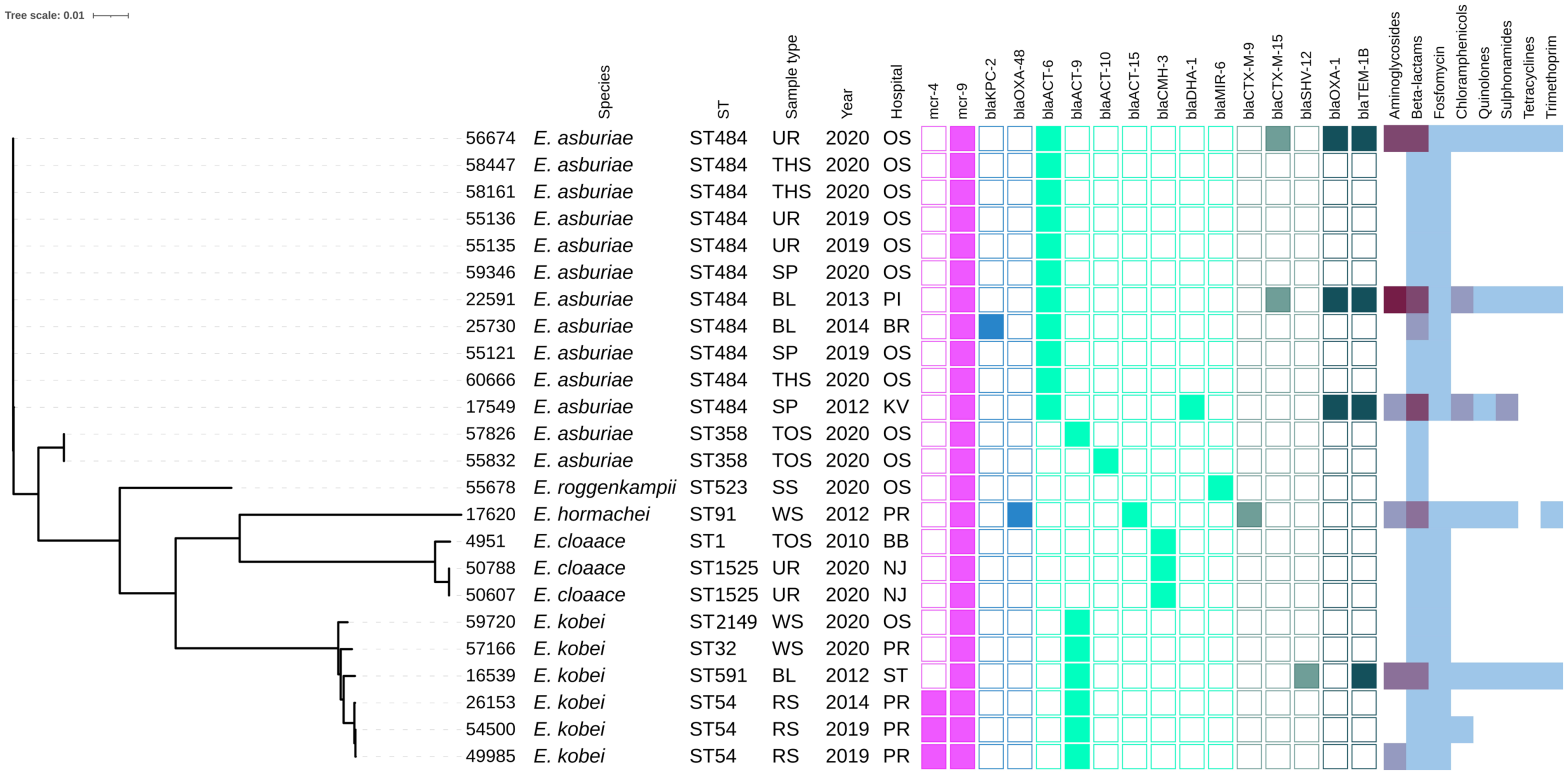
Phylogenetic tree of *Enterobacter* spp. isolates of Czech clinical origin. The metadata specify the species (Species); sequence type (ST), type of sample (Sample type): urine (UR), throat swab (TSH), sputum (SP), blood (BL), tonque swab (TOS), skin swab (SS), wound swab (WS), rectal swab (RS); year of isolation (Year) and city where is the hospital related to the isolate recovery (Hospital): Ostrava (OS), Pribram (PI), Brno (BR), Karlovy Vary (KV), Prague (PR), Brno-Bohunice (BB), Novy Jicin (NJ), Strakonice (ST). The color squares represent presence (full square) or absence (empty square) or respective antibiotic resistance genes divided to genes encoding resistance to colistin (pink), carbapenemases (blue) and other beta-lactamases (turquoise, see legend Figure 1). The heat map in the last section indicated the amount of antibiotic resistance genes carried by the respective isolate in specified category of antibiotics from zero (white) to maximum of five (dark purple).

The *mcr-1*-positive *E. coli* isolates belonged to 26 various STs of which *E. coli* ST744 was the most common (10/44). Fifteen *E. coli* isolates were assigned to ST88 (*n* = 3), ST538 (*n* = 3), ST1011 (*n* = 3), ST69 (*n* = 2), ST162 (*n* = 2), and ST453 (*n* = 2), while the remaining 19 isolates belonged to unique STs (Supplementary Table S1 and Figure 1). Four *K. pneumoniae* isolates carrying *mcr-1* were assigned to four different STs (ST147, ST231, ST290, and ST726) and one *C. freundii* isolate with *mcr-9* belonged to ST18. *Enterobacter* spp. with *mcr-9* belonged predominantly to *E. asburiae* of two different STs, including ST484 (*n* = 11) and ST358 (2) (Figure 2). Six isolates of *E. kobei* belonged to ST32 (*n* = 1), ST2149 (*n* = 1), ST591 (*n* = 1), and ST54 (*n* = 3). All *E. kobei* ST54 isolates originated from patients in a single hospital and carried *mcr-4* apart from *mcr-9*. Two remaining isolates were identified as *E. cloacae* ST1525 and were obtained from one patient.

Most *mcr-1-*positive isolates carried genes (Supplementary Table S1) conferring resistance to aminoglycosides (36/48), macrolides (42/48), sulphonamides (36/48), tetracycline (33/48) and trimethoprim (31/48). Additionally, 44 *mcr-1*-positive isolates harbored genes for resistance to narrow-spectrum beta-lactams, including *bla*_TEM-1B_ (*n* = 27), *bla*_TEM-135_ (*n* = 4) and *bla*_TEM-32_ (*n* = 3). In two isolates, AmpC beta-lactamase genes *bla*_CMY-2_ or *bla*_DHA-1_ were detected, while in three isolates, the ESBL genes *bla*_CTX-M-1_ (*n* = 1), *bla*_CTX-M-27_ (*n* = 2) were found. All four *mcr-1*-positive *K. pneumoniae* isolates carried *fosA*, *oqxA*, *oqxB* and *bla*_SHV_ genes.

On the other hand, the majority of *mcr-9*-positive isolates contained resistance genes to beta-lactams (*n* = 25) and fosfomycin (*n* = 22). Specifically, the *E. asburiae* ST484 isolates carried the *bla*_ACT-6_ gene, encoding the intrinsic AmpC beta-lactamase, while the *E. asburiae* ST358 isolates carried *bla*_ACT-9_ or *bla*_ACT-10_ variants (Figure 2). The two *E. cloacae* ST1525 isolates harbored only the *bla*_CMH-3_ gene, encoding the chromosomal AmpC. The ST54 *E. kobei* isolates carried the *bla*_ACT-9_ and *fosA* genes. Moreover, ESBL (*bla*_CTX-M-9_, *bla*_CTX-M-15_) and AmpC beta-lactamase (*bla*_DHA-1_, *bla*_CMY-117_) were identified in three and two *mcr-9*-positive isolates, respectively. One *E. hormaechei* ST91 isolate carried carbapenemase gene *bla*_OXA-48_ on IncL plasmid, while the single *Citrobacter freundii* ST18 isolate carried five beta-lactamase genes, including carbapenemase-encoding gene *bla*_KPC-2_ carried by IncR plasmid.

Chromosomal mutations to fluoroquinolones (*acrR*), cephalosporins (*ompK36*) and carbapenems (*ompK37*) were identified in four *K. pneumoniae* isolates (Supplementary Table S1). In contrast with the susceptibility testing, three of these *K. pneumoniae* isolates were resistant to fluoroquinolones and only one to cephalosporins. Twenty-five *E. coli* isolates carried at least one of four mutation variants, including *gyrA, gyrB, parC*, and *parE* for resistance to quinolones, and 13 *E. coli* isolates carried quinolone-resistance genes *qnrB* (*n* = 5) or *qnrS* (*n* = 8) (Supplementary Table S1) while MIC profiles showed quinolone resistance in 31 isolates. Nine different types of mutations in *pmrA/pmrB* genes associated with colistin resistance were also found in 11 *E. coli* isolates (Supplementary Table S1).

### Phylogenetic analysis of *Escherichia coli* ST744

**Figure 3:**
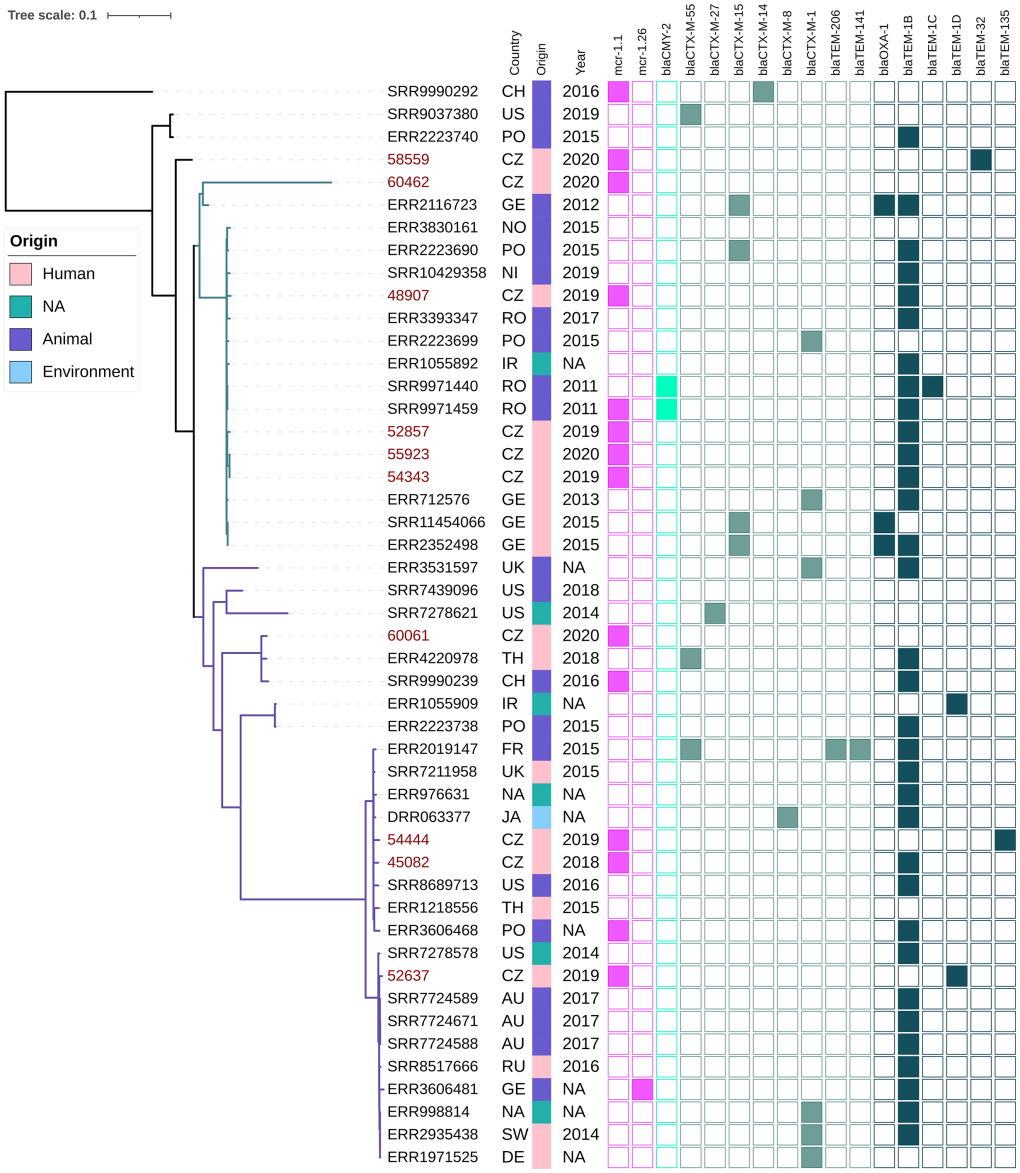
Phylogenetic tree of Czech clinical *E. coli* ST744 isolates with selected sequences from global collection. The red labels indicate isolates coming from this study. The metadata specifies country of origin (Country): China (CH), The United States (US), Poland (PO), Czech Republic (CZ), Germany (GE), Norway (NO), Nigeria (NI), Romania (RO), United Kingdom (UK), Thailand (TH), Ireland (IR), France (FR), Ukraine (UK), Australia (AU), Russia (RU), Switzerland (SW), not available (NA), the source of origin (Source) and the year of isolation (Year). The colour squares represent presence (full square) or absence (empty square) or respective antibiotic resistance genes divided to genes encoding resistance to colistin (pink), carbapenemases (blue) and other beta-lactamases (turquoise, see legend Figure 1).

Phylogenetic analysis of ST744 isolates from our collection (*n* = 10) along with other 38 closely related genomes from public resources showed the formation of two major clades; four isolates were considered as outliers (Figure 3). The first major clade (samples 60462-ERR2352498, green branch) comprised 17 mostly European isolates from animals and humans, and was further divided into two subclades. The second dominant clade (samples ERR3531597 – ERR1971525, violet branch) was composed of 27 cosmopolitan isolates originating from various sources, and was divided into several smaller subclades. Isolates belonging to different major clades showed a variable number of pairwise SNP differences against each other, ranging from 600 up to 3,000. In both major clades, there were apparent clusters of isolates from humans and animals, exhibiting a few dozen of SNPs from each other (Supplementary Table S2). Isolates from our collection were scattered across the tree, five of them belonged to the first clade, four to the second clade, while one sample was an outlier. Our clinical samples 48907, 52857, 55923 and 54343, belonging to the first clade, showed 46–58 SNP differences from three clinical samples from Germany, and 39–49 SNP differences from the two Romanian (RO) isolates from poultry that carried *bla*_CMY-2_. One of the RO isolates also carried the *mcr-1.1* gene that was borne by all isolates from our Czech clinical collection. Those RO isolates were also closely related to three clinical isolates from Germany (exhibited <20 SNP differences from each other). Isolate 60061 from the Czech collection clustered with a clinical isolate from Thailand (110 SNPs) and a Chinese isolate from a pig (128 SNPs). Notably, the Swiss isolate also carried *mcr-1.1*. Our isolates 45082 and 54444 were related to another clinical isolate from the United Kingdom (66 and 73 SNPs) and also to an environmental isolate from a river in Japan (73, and 80 SNPs, respectively). Isolate 52637 from our collection showed the least SNP counts against three Australian isolates from gulls (36–37 SNPs) and three clinical isolates, one from Switzerland (36 SNPs), and another from Germany (39 SNPs), and Russia (42 SNPs).

### Structure of *mcr-1*-carrying plasmids

The *mcr-1* gene was located predominantly on 33 kb IncX4 plasmids (34/48). Six complete plasmids from *E. coli* and *K. pneumoniae* obtained by long-read sequencing (Table 1) showed a high level of nucleotide similarity (>99.9%) to each other as well as to plasmids from raw meat from Czech retails (Zelendova et al., 2021; Supplementary Figure S1). The *mcr-1* gene was bordered by a hypothetical protein and a PAP2 transmembrane protein, which is the typical genetic surrounding for *mcr-1* gene within IncX4 plasmids (Zelendova et al., 2021).

The *mcr-1* was also carried by ~60 kb IncI2 plasmids (*n* = 8). Plasmid pMCR1-53288 originating from *E. coli* ST538 from urine obtained by MinION sequencing shared high sequence similarity (>98%) with several plasmids available in the GenBank database, including pMCR_1884_C3 and pMCR_1138_A1 from *C. braakii* and *E. coli* ST162, respectively, isolated from imported meat sold in Czech retails (Zelendova et al., 2021; Supplementary Figure S2). The *mcr-1* region was inserted downstream of the *nikB* gene, encoding a DNA topoisomerase III, as observed in other IncI2 *mcr-1*-positive plasmids like pMCR_1884_C3. No other resistance genes were located on IncI2 plasmids.

**Figure 4:**
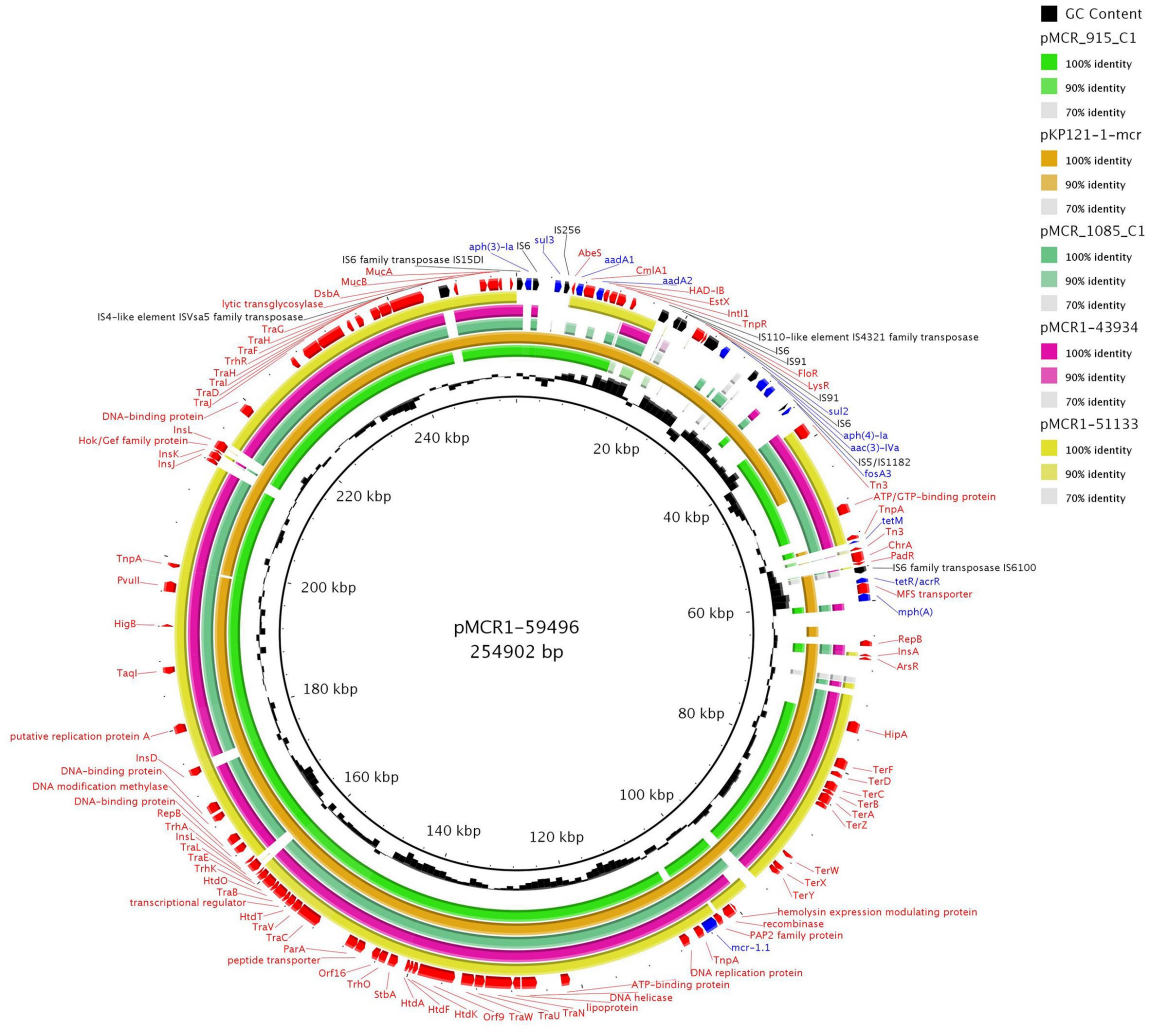
BRIG comparison of *mcr-1.1*-encoding IncHI2/ST4 plasmids. The plasmid pMCR1-59496 from *K. pneumoniae* ST726 identified in our study from urine sample (OP950836) was used as a reference. Two other plasmids originated from our collection, including pMCR1-43934 from *E. coli* ST8186 from tonsil swab sample and pMCR1-51133 from *E. coli* ST117 from a urine sample. The sequence alignment includes pMCR_915_C1 (MT929284.1) and pMCR_1085_C1 (MT929286.1) from *E. coli* recovered from raw turkey meat imported to the Czech Republic from Poland and one of plasmid pKP121-1-mcr (CP031850.1) from *K. pneumoniae* ST2570 from human blood in China.

From our collection, six *Enterobacterales* isolates were found to harbor IncHI2 plasmids with the *mcr-1* gene. The complete sequence of three *mcr-1*-positive IncHI2 plasmids, pMCR1-59496, pMCR1-43934, and MCR1-51133, was determined using MinION technology. BlastN analysis showed that all sequenced IncHI2 plasmids, ranging from ~225 to ~255 kb in size, belonged to ST4 and were closely related (coverage 80–99%, identity 99%) to each other (Figure 4), as well as to other *mcr-1*-carrying IncHI2 plasmids, like pMCR_915_C1 and pMCR_1085_C1 from *E. coli* recovered from imported meat (Zelendova et al., 2021), and plasmid pKP121-1-mcr (Ruan et al., 2019) of human clinical origin from China. All IncHI2 plasmids contained regions for conjugative transfer (*htd*, *orf*, *tra* genes) and plasmid maintenance (*par* gene). Additionally, IncHI2 plasmids carried tellurium resistance genes in two clusters, including *terZABCDEF* and *terXYW* (except p56099). In all InHI2 plasmids, characterized during this study, the *mcr-1* gene was inserted downstream of the *terY2* gene, as observed in other IncHI2 plasmids like pMCR_1085_C1. Similar to pMCR_1085_C1, the *mcr-1* gene was bounded by an IS*Apl1* element and PAP2 transmembrane protein (Zelendova et al., 2021). All IncHI2 *mcr-1*-positive plasmids exhibited at least one MDR region, which ranged in size from 950 to 36,097 bp (Figure 4).

### Structure of *mcr-4-*encoding plasmids

*mcr-4* was located on ColE10 plasmids in three *E. kobei* ST54 isolates. Plasmid pMCR4-26153 of size 12,808 bp recovered from a rectal swab of a patient in the Czech Republic was identical (100% coverage, 100% identity) to pIB2020_ColE_MCR (Marchetti et al., 2021) from *E. kobei* ST54 strain from a rectal swab of a 56 years old male patient hospitalized in 2019 in Italy (Supplementary Figure S3).

### Structure of *mcr-9.1*-carrying elements

Out of the 25 *mcr-9.1*-positive isolates, eight were characterized by MinION technology. Among the latter isolates, three carried the *mcr-9.1* allele on IncHI2 plasmids (Table 1) while, in the five remaining isolates, the *mcr-9*.*1* was found on the chromosome. Plasmid pMCR9-57185 originated from *C. freundii* ST18 recovered from a rectal swab, while pMCR9-16539 was obtained from *E. kobei* ST591 from blood and pMCR9-17620 came from *E. hormaechei* ST91 recovered from a wound swab.

Following the IncHI2 pDLST scheme, plasmids pMCR9-57185 and pMCR9-16539 were typed as ST1, while pMCR9-17620 was assigned to ST17. All plasmids exhibited closely related sequences (>89% coverage, 99.99% identity) to other *mcr-9.1*-positive IncHI2 plasmids (Supplementary Figure S4), like p49790_MCR from an *E. hormaechei* isolate recovered previously from Czech hospitals (Bitar et al., 2020). Similar to p49790_MCR, the *mcr-9.1* was inserted upstream of the *pcoS* gene (encoding a membrane protein for resistance to copper), in all IncHI2 plasmids like p49790_MCR. Additionally, in plasmids pMCR9-57185 and pMCR9-16539, the *mcr-9.1* gene was bounded by an IS element (upstream) and an ORF (downstream), encoding a cupin fold metalloprotein, followed by IS*26*. However, in plasmid pMCR9-17620, an IS*1* was found downstream of *mcr-9.1*. Furthermore, IncHI2 plasmids contained at least one MDR region, including genes for resistance to aminoglycosides, tetracyclines, trimethoprim, chloramphenicol, sulfonamides, quinolones, and/or macrolides (Table 1). Moreover, IncHI2 plasmids carried tellurium resistance genes (*terZABCDEF*) commonly associated with this plasmid family, and genes conferring arsenic resistance (*arsCBRH*).

**Figure 5:**
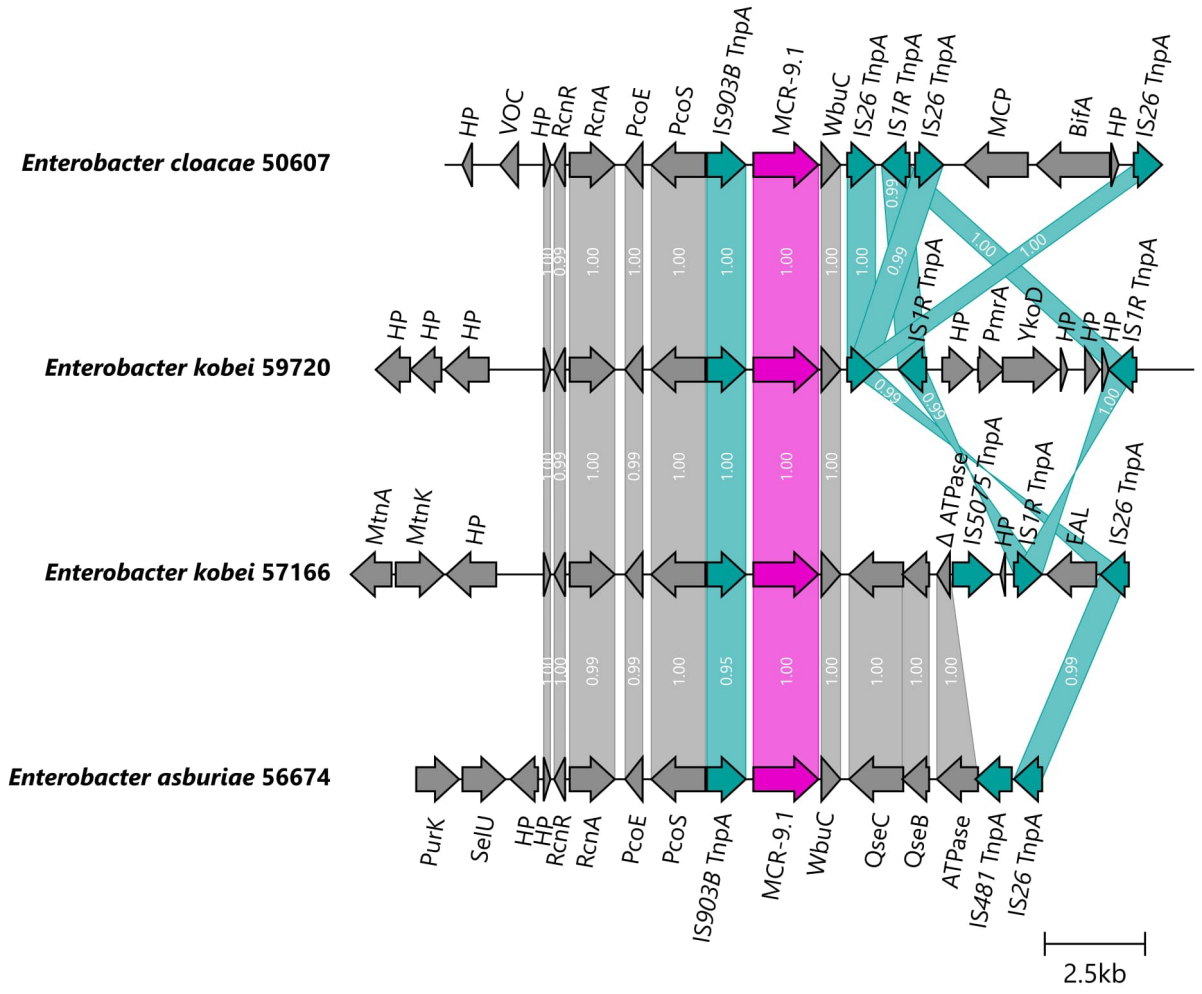
Genetic surroundings of *mcr-9.1* gene located on chromosomes in *Enterobacter*. The linearized coding sequences of MCR-9.1 region of four isolates were compared using clinker with identity threshold 90%. The MCR-9.1 (pink) surrounded by mobile genetic elements (turquoise) and other coding sequences (gray) in isolates 50607 and 59720 formed a region corresponding to the *mcr-9* region observed in IncHI2/ST1 plasmids. On the other hand, 57166 and 56674 isolates contained downstream sequence similar to the previously described *mcr-9* environment in *E. asburiae* (AP022628). White numbers in links correspond to the similarity of coding sequences.

The *mcr-9.1* gene was integrated into the chromosomes of four *E. cloacae* complex isolates obtained by long-read assembly. The upstream genetic surroundings were identical in all isolates, consisting of *mcr-9.1*, IS*903B*, *pcoS*, *pcoE*, *rcnA*, and *rcnR* genes, while the downstream sequences differed. In isolates 50607 and 59720, the *mcr-9.1* was followed (downstream) by *wbuC*, IS*26* and IS*1A* forming a region *rcnR*-*rcnA*-*pcoE*-*pcoS*-IS*903B*-*mcr-9.1*-*wbuC*-IS*26* identical to the respective region of the plasmid pMCR9-57185. On the other hand, the downstream environment of *mcr-9.1* in isolates 56674 and 57166 consisted of *wbuC*, *qseC*, *qseB* and ATPase ORF similar to the corresponding region in the chromosome of a Japanese human isolate *Enterobacter asburiae* A2563 (AP022628), visualized in Figure 5.

### Horizontal transfer of *mcr* gene

Resistance to colistin associated with *mcr-1* was transferred to recipient *E. coli* laboratory strains *via* conjugation in the majority of *E. coli* isolates (38/44, 86%) and in all isolates of *K. pneumoniae* (4/4, 100%). The most frequently transferred plasmid harboring *mcr-1* included 33 kb plasmid IncX4 (31/34, 91%), followed by 55 kb IncI2 (8/8, 100%) and 220 kb IncHI2 (4/6, 67%) plasmids. IncHI2 plasmid with *mcr-9* was transferred *via* conjugation in one *E. asburiae* isolate. Also, ColE10 plasmid carrying *mcr-4* was not transferred *via* conjugation, since this plasmid family does not contain any transfer region.

## Discussion

Within this study, we performed a surveillance of *mcr*-encoding genes among colistin-resistant *Enterobacterales* collected from Czech hospitals between 2009 and 2020. Our findings indicated a low prevalence of *mcr* genes among colistin-resistant isolates, with a slightly increasing prevalence during the study period. However, the prevalence of *mcr*-positive isolates may be overestimated since our collection was composed only of colistin-resistant isolates and did not include the bacterial population susceptible to colistin. Moreover, another study limitation is the fact that isolates from retrospective sampling from the period 2009–2017 were obtained during various surveillance programmes at the National Institute of Public Health. As these programmes were focused mainly on *Klebsiella* sp., invasive *E. coli* or they were targeting MDR strains, the data of *mcr* prevalence from this period needs to be interpreted with caution.

Other studies from Europe have reported a low prevalence of colistin-resistant isolates and of *mcr*-positive *Enterobacterales*. The Czech study by Tkadlec et al. (2021) found *E. coli* with *mcr-1* gene in only 0.21% (4/1922) of fecal samples from hospitalized patients. A study from Switzerland reported that the fecal carriage rate of colistin-resistant (MIC value >2 mg/L) *Enterobacterales* was 1.5% for healthy people and 3.8% for primary care patients (Zurfluh et al., 2017). Additionally, in Finland, only one *mcr-1*-positive *E. coli* was characterized from fecal samples collected from 176 healthy volunteers (Gröndahl-Yli-Hannuksela et al., 2018). In Spain, the overall prevalence of colistin resistance in clinical isolates of *Enterobacterales* was 0.67% and *mcr-1* was detected only in *E. coli* (0.15%) (Prim et al., 2017). Similar prevalence levels were observed for Romagna, Northern Italy, where the prevalence of colistin-resistant isolates among human *Enterobacterales* was 0.5% and the *mcr-1* gene was found in 0.14% *E. coli* isolates (Del Bianco et al., 2018). On the other hand, higher percentages of *mcr-*carrying isolates have been reported in some areas outside Europe. Giani et al. (2018) reported a high proportion (38.8%) of *mcr-1* carriers among healthy children (129/337) from Bolivia. Furthermore, in Chinese hospitals across 24 provincial capital cities and municipalities, human carriage of *mcr-1*-positive *E coli* was identified in 644 (14.3%) of 4,498 samples in 2016 (Wang Y. et al., 2020). However, different methodological approaches, study designs and sample types (e.g., selective cultivation on colistin-supplemented media, targeting isolates despite their susceptibility profiles, PCR detection of *mcr* genes in either total enterobacterial gut microbiota or directly in a clinical sample) significantly limit the comparison of prevalence data between the studies. Moreover, the discrepancy in the prevalence of *mcr* carriers between studies and geographical regions underlines the other factors, like antibiotic use and stewardship protocols, contributing to the emergence and spread of colistin-resistant isolates.

In this study, the majority of isolates carried *mcr-1* followed by *mcr-9* and *mcr-4* variants. Most *mcr-1* carriers were *E. coli* and the gene was present in 23% of all resistant isolates of this species, which is in agreement with findings of previous study (Zelendova et al., 2021). Most of the *mcr-9.1*-carrying isolates in our study belonged to *Enterobacter* spp. Previous studies have shown that the *mcr-9* gene is commonly associated with isolates belonging to the *Salmonella* and *Enterobacter* genus (Bitar et al., 2020; Liao et al., 2022; Zhang et al., 2022). Interestingly, the low resistance levels to colistin of MCR-9-producing *Enterobacter* isolates have been reported (Bitar et al., 2020). This observation may explain the unnoticed spread of those isolates in Czech hospitals. Remarkably, 96% of isolates (70/73) carried AmpC/ESBL or carbapenemases, raising the concern that the spread of *mcr-*carrying isolates might also be related to the use of other antimicrobial agents, including clinically important beta-lactams.

MLST revealed the presence of *mcr* genes in various STs of *E. coli*, *K. pneumoniae*, and *Enterobacter* sp., highlighting the significant impact of horizontal gene transfer in the spread of colistin resistance determinants *via* plasmids. Additionally, phylogenetic analysis uncovered the association of *mcr* genes with specific clones, like *E. kobei* ST54, which has been previously reported to produce MCR-4.3 from clinical samples recovered in Italy (Marchetti et al., 2021). Of note, these observations underline the important role of traveling across borders, that has contributed to the spread of MDR bacteria.

Finally, analysis of *mcr*-carrying plasmid sequences showed the presence of *mcr-1*, mainly on IncX4 replicons, but also on IncI2 and IncHI2 plasmids. These findings are in agreement with the previously published data, showing the emergence of *mcr* genes on the specific Inc groups of plasmids that were characterized from *Enterobacterales* recovered from different sources, including animals, food and humans (Xavier et al., 2016; Zurfluh et al., 2017; El Garch et al., 2018; Bitar et al., 2020; Li et al., 2020; Marchetti et al., 2021; Zelendova et al., 2021). Furthermore, our experiments demonstrated a high efficiency of conjugative transfer of *mcr-1*-carrying IncX4 plasmids. Also, the conjugative transfer of IncHI2 plasmids carrying *mcr-1* or *mcr-9* was confirmed. Thus, the horizontal transfer of plasmid-mediated *mcr* genes represents an important risk factor for public health since colistin is considered as one of the last-resort antibiotics for the treatment of serious infections in human medicine. Therefore, studying the spread of MDR pathogens is vital for analysis of transmission pathways and risk factors for public health.

The prospective epidemiological survey performed in this study brought the first information on the plasmid-mediated dissemination in the Czech Republic and showed that a surveillance system is essential to monitor the diffusion of plasmid mediated colistin resistance.

## Data availability statement

The datasets presented in this study can be found in online repositories. The names of the repository/repositories and accession number(s) can be found below: https://www.ncbi.nlm.nih.gov/genbank/, PRJNA772899.

## Ethics statement

Ethical review and approval was not required for the study on human participants in accordance with the local legislation and institutional requirements. Written informed consent for participation was not required for this study in accordance with the national legislation and the institutional requirements.

## Author contributions

MZ performed laboratory work, data analysis, and prepared the manuscript. CP performed data analysis and prepared the manuscript. PS performed laboratory work and helped with the manuscript preparation. MM performed bioinformatic analyses of whole-genome sequencing data. JP conducted MinION sequencing and helped with plasmid analysis. KN contributed on figure preparation. VJ, KP, and HZ provided the samples. IJ performed whole-genome sequencing. MD supervised the project, performed data analysis, and revised the manuscript. All authors discussed the results. Members of the surveillance network provided the clinical isolates and metadata obtained during the standard microbiological testing in their laboratories.

## Working group for monitoring of antibiotic resistance

Vaclava Adamkova, First Faculty of Medicine and University Hospital, Charles University, Prague; Natasa Bartonikova, Bata’s Hospital, Tamara Bergerova, Faculty of Medicine and University Hospital in Plzen, Charles University, Plzen; Marie Bohackova, Hospital in Chrudim, Chrudim; Czysova Erika, Hospital in Sumperk, Sumperk; Josef Cermak, Health institute Usti nad Labem, Kladno; Martina Curdova, Military Hospital Praha, Daniela Fackova, Liberec Regional Hospital, Liberec; Linda Drabkova, University Hospital in Brno, Brno; Lenka Dvorakova, Masaryk Hospital in Usti nad Labem, Usti nad Labem; Galina Eliasova, Regional Hospital Kladno, Kladno; Vladimir Fibiger, Hospital and Polyclinic Ceska Lipa, Ceska Lipa; Marian Glasnak, Rudolf and Stefania Benesov Hospital, Benesov; Vera Haskova, Health institute Usti nad Labem, Horovice; Gabriela Hedvicakova, Hospital in Semily, Semily; Blanka Horova, Bulovka University Hospital, Prague; Eva Chmelarova, AGELLAB, Ostrava-Vitkovice; Jan Kubele, Hospital Na Homolce, Prague; Eva Jechova, Thomayer University Hospital, Prague; Petr Jezek, Regional Hospital in Pribram, Pribram; Helena Jordakova, University Hospital in Kralovske Vinohrady, Prague; Jana Jurankova, SPADIA LAB, Brno; Miloslava Kocianova, SYNLAB, Prague; Ivana Kohnova, AGEL Prostejov Hospital, Prostejov; Dana Krckova, IFCOR-99, Brno; Eva Krejci, Health Institute in Ostrava, Ostrava; Hana Kremeckova, Hospital in Kyjov, Kyjov; Alice Kucharova, Hospital in Tabor; Katerina Laskafeldova, AGEL Laboratory, Novy Jicin; Jiri Malina, AeskuLab Hadovka, Prague; Eva Martinkova, DIA-GON MP, Cheb; Monika Mazurova, Hospital Usti nad Labem, Zamberk; Marian Mednansky, Hospital in Havlickuv Bod, Havlickuv Brod; Eliska Miskova, Hospital in Trebic, Trebic; Lenka Nanakova, Hospital in Hodonin, Hodonin; Helena Nedvedova, Hospital in Klatovy, Klatovy; Otakar Nyc, University Hospital in Motol, Charles University, Prague; Blanka Ochvatova, SPADIA LAB, Ostrava; Pavla Paterova, University Hospital in Hradec Kralove, Hradec Kralove; Zdena Pitakova, Hospital in Vyskov; J. Podrouzkova, Sang Lab, Karlovy Vary; Miroslava Prejzkova, Synlab, Chomutov; Renata Pribikova, Hospital in Litomerice; Blanka Puchalkova, Hospital in Karlovy Vary, Karlovy Vary; Jana Repiscakova, Hospital in Uherske Hradiste, Uherske Hradiste; Zuzana Semerakova, SPADIA LAB, Prague; Helena Skacani, Hospital in Jihlava, Jihlava; Marketa Skruzna, Institute of Clinical and Experimental Medicine, Prague; Marie Smolikova, Hospital in Jicin, Jicin; Martina Sosikova, Silesian Hospital in Opava, Opava; Michal Stanek, Hospital in Znojmo, Znojmo; Alena Steinerova, CITYLAB, Prague; Petra Safarova, Laboratory of Medical Microbiology, Pardubice; Lenka Semberova, Czech Laboratory, Prague; Eva Simeckova, Hospital in Strakonice, Strakonice; Ljuba Suchmanova, Health institute Usti nad Labem, Plzen; David Sus, Hospital in Ceske Budejovice, Ceske Budejovice; Renata Tejkalova, University Hospital of St. Anna, Masaryk University, Brno; Jan Tkadlec, Hospital in Vsetin, Vsetin; Lenka Unuckova, Hospital in Kolin, Kolin; Danuta Urbusova, AGEL Laboratory, Trinec; Vera Kurkova, Hospital in Pisek, Pisek; Denisa Vesela, Hospital in Jindrichuv Hradec, Jindrichuv Hradec; Eva Vesela, Hospital in Nachod, Nachod; Eva Vitova, Hospital in Trutnov, Trutnov; Eva Zalabska, Hospital in Pardubice, Pardubice; Dana Zamazalova, Hospital in Nove Mesto Na Morave, Nove Mesto Na Morave; Roman Zaruba, Hospital in Most, Most; Ilona Zemanova, VIDIA DIAGNOSTIKA, Prague.

## Funding

This project was funded by projects of Czech Health Research Council NV18-09-0060 and NU20J-09-0040, the Internal Grant Agency 205/2022/FVHE and partially Ministry of Health, Czech Republic – conceptual development of research organization (FNBr, 65269705).

## Conflict of interest

The authors declare that the research was conducted in the absence of any commercial or financial relationships that could be construed as a potential conflict of interest.

## Publisher’s note

All claims expressed in this article are solely those of the authors and do not necessarily represent those of their affiliated organizations, or those of the publisher, the editors and the reviewers. Any product that may be evaluated in this article, or claim that may be made by its manufacturer, is not guaranteed or endorsed by the publisher.

## Footnotes

^1^ https://cge.cbs.dtu.dk/

^2^ https://jgi.doe.gov/data-and-tools/software-tools/bbtools/bb-tools-user-guide/

^3^ www.ncbi.nlm.nih.gov/BLAST

^4^ www.bioinformatics.org/sms/

^5^ http://enterobase.warwick.ac.uk/

^6^ https://github.com/matejmedvecky/anthraxdiversityscripts

^7^ https://inkscape.org/cs/

^8^ https://github.com/tseemann/snp-dists
