# Supplementary figures and images for: Plasmid-mediated colistin resistance among human clinical Enterobacterales isolates: national surveillance in the Czech Republic
Source: Front Microbiol. 2023 Apr 27;14:1147846. doi: 10.3389/fmicb.2023.1147846 (PMC10174314; doi:10.3389/fmicb.2023.1147846)

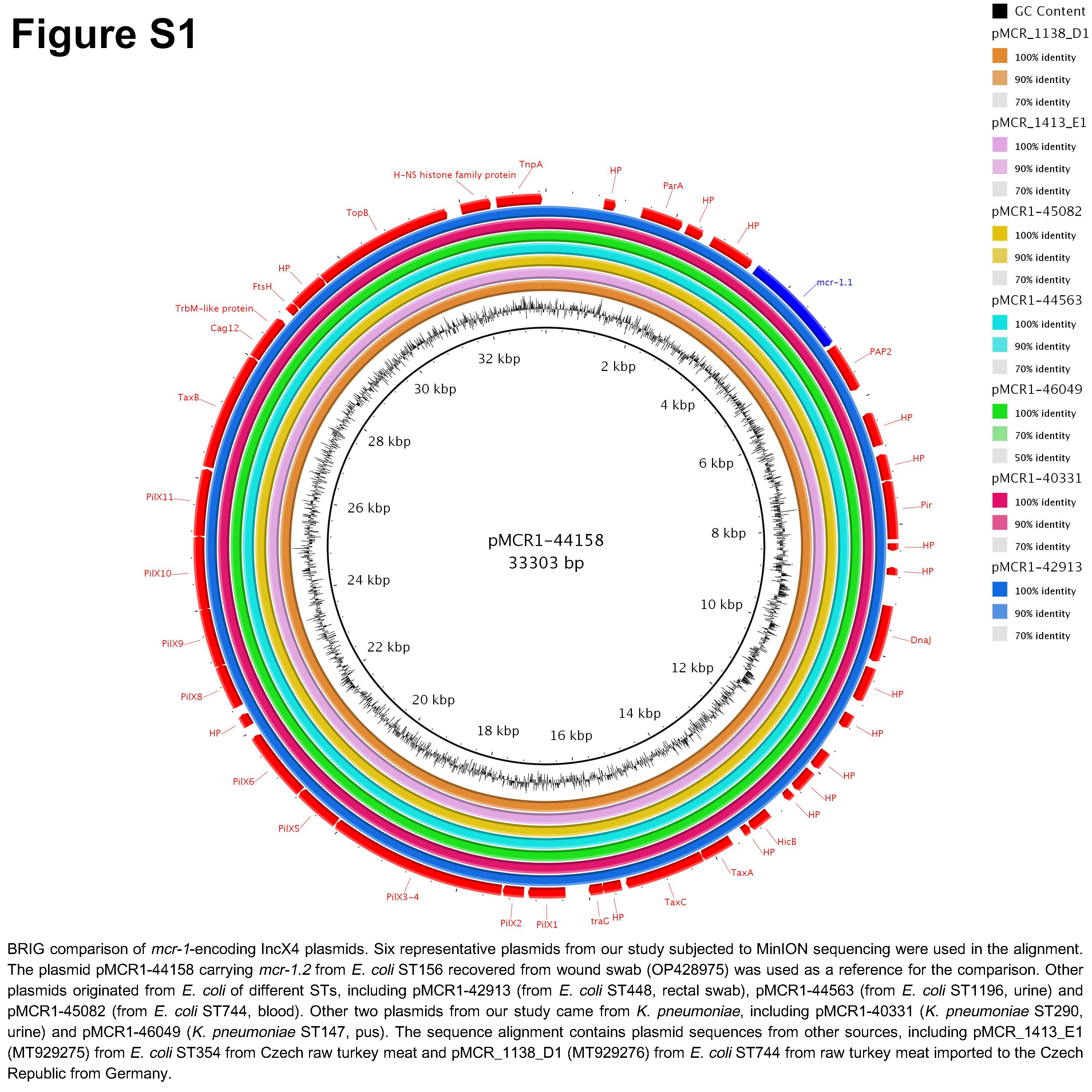

Supplement: Supplementary file 3 [file Image_1.jpeg]

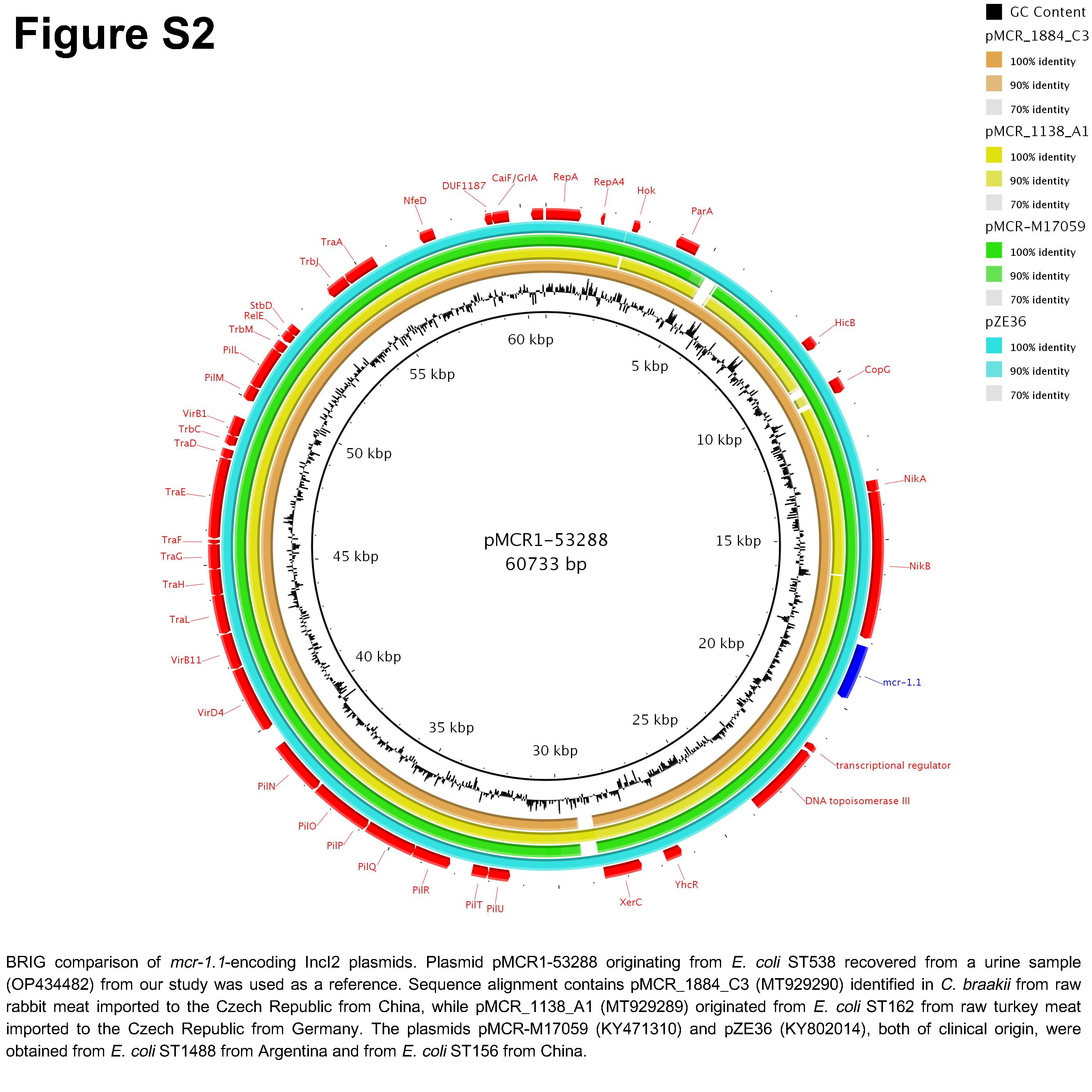

Supplement: Supplementary file 4 [file Image_2.jpeg]

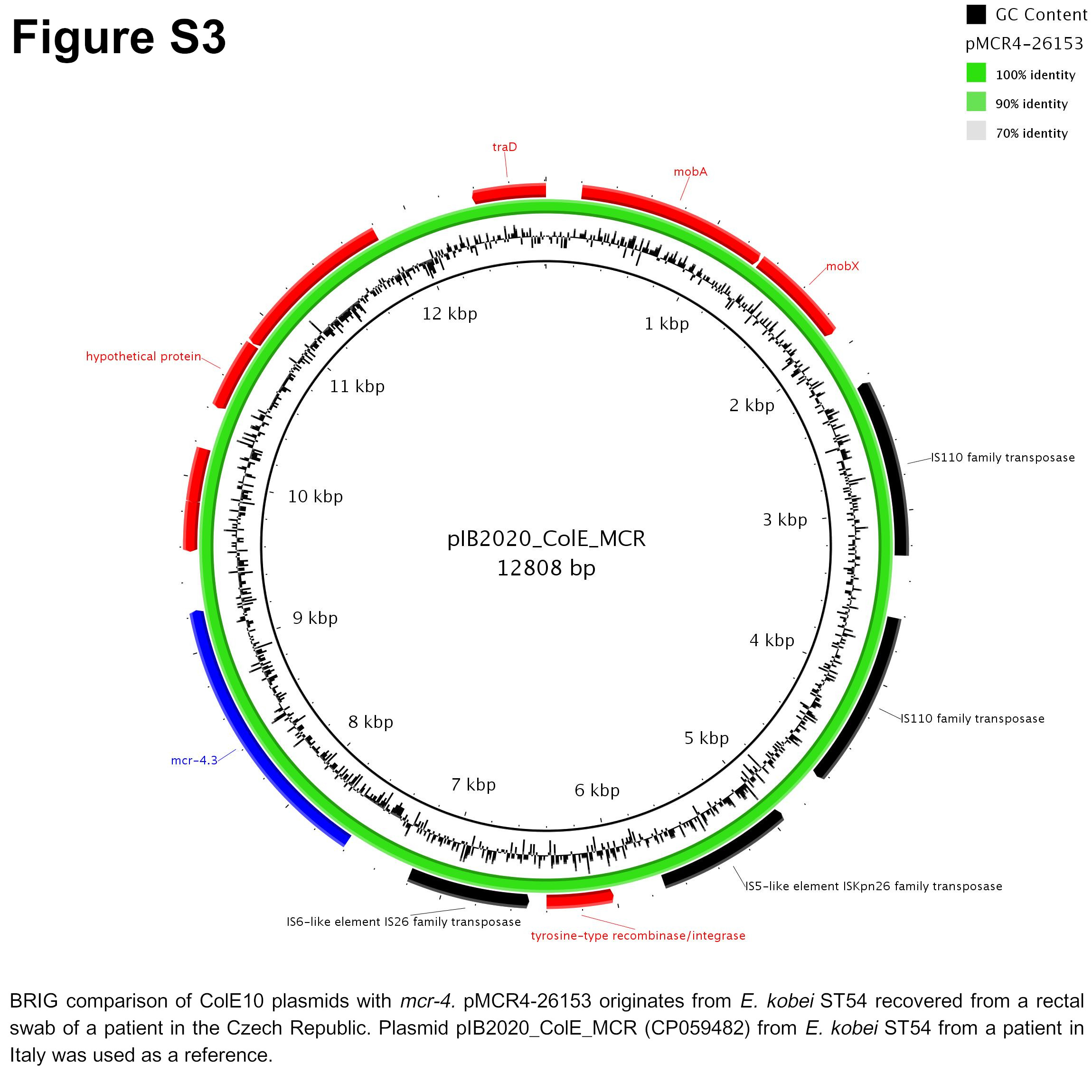

Supplement: Supplementary file 5 [file Image_3.jpeg]

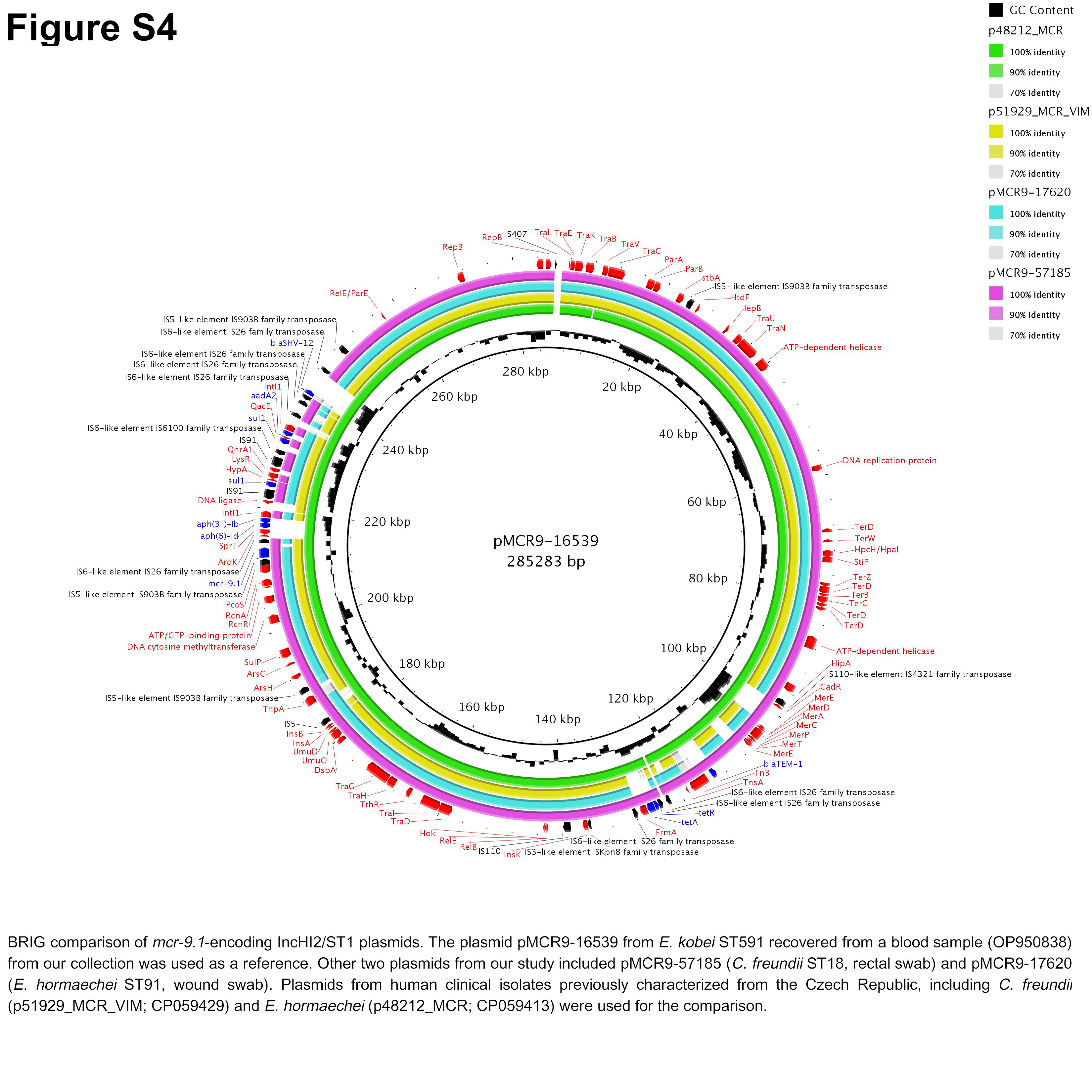

Supplement: Supplementary file 6 [file Image_4.jpeg]
